# Job Satisfaction and Psychological Distress Among Healthcare Professionals

**DOI:** 10.1192/j.eurpsy.2025.2018

**Published:** 2025-08-26

**Authors:** M. Theodoratou, H. Vavatsikou, E. Denazi, I. Papathanasiou

**Affiliations:** 1 Social Sciences, Hellenic Open University., Patras, Greece; 2 Psychology, Neapolis University Pafos, Pafos, Cyprus; 3 Psychology, National Kapodistrian University of Athens, Athens, Greece

## Abstract

**Introduction:**

Job satisfaction and psychological distress are pivotal elements in the overall well-being of healthcare professionals, whose work environment is inherently challenging and stressful. High levels of job satisfaction are essential for the health, motivation, and performance of the workforce, and for ensuring high-quality patient care and the effective functioning of the healthcare organization. Conversely, psychological distress among healthcare workers can result in burnout, a reduction in the quality of care provided, and an increase in turnover rates. This study examines the complex factors influencing job satisfaction and psychological distress, with the aim of providing a comprehensive understanding that can inform the development of interventions and policy changes within healthcare settings.

**Objectives:**

The purpose of this study is to explore the factors that contribute to job satisfaction and psychological distress among medical and nursing staff. By identifying conditions that lead to increased satisfaction or distress, the research seeks to propose actionable strategies to improve the working environment and reduce psychological strain among healthcare workers.

**Methods:**

This research employed a quantitative approach utilizing a specifically designed questionnaire that incorporated the DASS21 (Depression, Anxiety, and Stress Scale) and the K6+ scale for measuring psychological distress. The sample consisted of 132 healthcare professionals, selected through the snowball sampling method. The questionnaire was based on a comprehensive review of the literature and included measures to assess job satisfaction, anxiety, stress, and psychological distress.

**Results:**

The findings indicate that the majority of healthcare workers perceive limited opportunities for job advancement and salary increases. Furthermore, communication within organizations is perceived as moderate, and employees report that their efforts are not adequately recognized or rewarded. These factors are associated with both job dissatisfaction and increased psychological distress, as measured by the DASS21 and K6+ scales.

**Conclusions:**

The findings of this study highlight the imperative for systemic reforms within healthcare organizations to enhance job satisfaction and mitigate psychological distress among healthcare personnel. It is recommended that strategies include the creation of more transparent pathways for career advancement, the fostering of a supportive and communicative work environment, and the implementation of recognition programs that validate and reward employee contributions. It would be beneficial for future research to investigate the long-term impact of such interventions in order to ascertain their efficacy in maintaining positive changes within healthcare settings.

**Disclosure of Interest:**

None Declared

